# The effects of the interaction between cyanidin‐3‐O‐glucoside (C3G) and walnut protein isolate (WPI) on the thermal and oxidative stability of C3G

**DOI:** 10.1002/fsn3.4309

**Published:** 2024-07-03

**Authors:** Daquan Wang, Haipeng Cui, Kaili Zong, Hongchao Hu, Yali Li, Jianting Yang

**Affiliations:** ^1^ Anhui Science and Technology University Chuzhou China

**Keywords:** cyanidin‐3‐O‐glucoside, degradation kinetics, interaction, walnut protein isolate

## Abstract

This study explores the interaction between cyanidin‐3‐O‐glucoside (C3G), a water‐soluble pigment known for its diverse functional activities, and walnut protein isolate (WPI) as a potential stabilizing agent. Given the inherent instability of C3G, particularly its limited application in the food industry due to sensitivity to thermal and oxidative conditions, this research study aims to enhance its stability. According to the results of the fluorescence quenching experiment, C3G can efficiently quench WPI's intrinsic fluorescence through static quenching. Structural exploration revealed that C3G bound WPI via hydrophobic interaction force, with the number of bound C3G molecules (*n*) almost equivalent to 1. The Δ*G* value denoting change in Gibbs free energy for C3G binding with WPI was −8.05 kJ/mol, which indicated that the interaction between C3G and WPI is spontaneous. Moreover, the conformational structures of WPI were altered by C3G binding with a decrease in α‐helix contents and an increase in β‐turn, β‐sheet, and random coil contents. The thermal degradation kinetics indicate that after interacting with WPI, the half‐life of C3G increased by 1.62 times and 1.05 times at 80 and 95°C, respectively. The results of the oxidation stability test showed that the presence of WPI effectively reduced the discoloration and degradation of C3G caused by oxidation, and improved the oxidation stability of C3G. This study's findings will help to clarify the interaction mechanism between C3G and WPI, and broaden C3G's application scope in the food processing field.

## Graphical abstract

Our study revealed the mode of interaction between mulberry anthocyanin (C3G) and walnut isolate protein (WPI) and the effect of WPI on the stability of C3G. The results showed that C3G interacted with WPI by means of static quenching and was a spontaneous heat‐absorbing reaction, with the main driving force being hydrophobic interactions. It was found that WPI prolonged the half‐life of C3G and improved the thermal and oxidative stability of C3G.

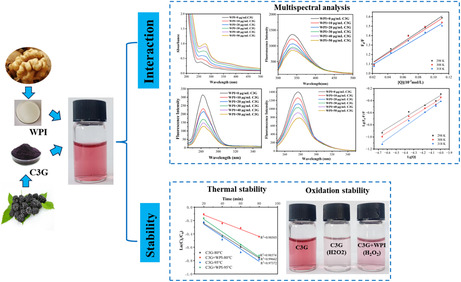

## 1 INTRODUCTION

Anthocyanin is an assortment of flavonoids found in many dark fruits, grains, and vegetables, such as mulberry, blueberry, black rice, and purple potato (Hao et al., 2021). Cyanidin‐3‐O‐glucoside (C3G) is widely distributed in nature (Ma et al., 2022). In addition, C3G has a strong ability to scavenge free radicals (Gong et al., 2021), with anticancer (Gui et al., 2023), anti‐inflammatory (Huo et al., 2023), antidiabetic (Go et al., 2021), enhancing immunity and vision (Mo et al., 2022) and other functional activities, and as a water‐soluble pigment, it has been widely used in the food industry. However, C3G is an unstable compound in aqueous solution. The stability of C3G is strongly reliant on environmental factors such as temperature (He et al., 2016a), light (Fang et al., 2020), pH, metal ions (Qiu et al., 2018), enzymes (Tong et al., 2020), etc. The application of C3G in food systems has poor stability during heat treatment and storage, which limits its application as a functional ingredient in food. Therefore, controlling the degradation of C3G has a significant impact on improving the stability of C3G and developing foods rich in plant‐active ingredients.

Currently, many studies have shown that proteins have an affinity for anthocyanins and can improve their stability through noncovalent interactions, by forming an anthocyanin–protein complex, anthocyanin molecules are encapsulated and protected in the complex, reducing their contact with external environments such as oxygen, light, and temperature, thereby delaying the oxidation and degradation of anthocyanins. As a natural antioxidant, protein can capture free radicals and slow down the oxidation reaction of anthocyanins. After the interaction between anthocyanins and proteins, proteins can protect anthocyanins from oxidation, thereby improving their stability (Attaribo et al., 2020; Chen et al., 2021; Fu et al., 2021). Noncovalent interactions include hydrophobic interactions, van der Waals forces, hydrogen bonding, and electrostatic attraction. For example, whey protein isolates can undergo noncovalent interactions with anthocyanins, significantly improving the thermal stability of C3G in the beverage model system through hydrogen bonding and van der Waals forces. Furthermore, adding preheated (50°C) whey protein isolate can make anthocyanin show the best color stability (Wang, Yang, et al., 2023). The interaction between bovine β‐lactoglobulin and anthocyanin extract from grape skin improves the thermal stability and oxidative stability of anthocyanins (He et al., 2016a). In addition, the interaction between silkworm pupa protein (SPP) and C3G through hydrophobic interactions effectively weakens the degradation of C3G and enhances its stability within a certain pH range (Attaribo et al., 2021). However, research on the protective effects of plant proteins on anthocyanins (such as food proteins) is limited.

Walnut protein is an excellent plant protein resource, which is widely used in food processing (Wang, Chen, et al., 2023). However, as the main component of walnut oil processing byproducts, the utilization rate of walnut protein is low, resulting in the waste of protein resources (Huang et al., 2022). Studies have shown that walnut protein can be used as a carrier protein to improve its sustained release behavior by hydrophobic interaction with curcumin, and also improve its water solubility and antioxidant activity (Moghadam et al., 2019). In addition, walnut protein interacts with polyphenols, which significantly improves the antioxidant activity of polyphenols and reduces surface hydrophobicity (Huang et al., 2022). However, there is little information on the binding interaction between anthocyanin and walnut protein and its effect on the stability of anthocyanin. Therefore, the purpose of this study is to characterize the interaction between walnut protein isolate (WPI) and C3G through ultraviolet (UV)–visible spectrum, fluorescence spectroscopy, synchronous fluorescence spectroscopy, and Raman spectroscopy techniques, and to evaluate the influence of WPI on the thermal and oxidative stability of C3G.

## 2 MATERIALS AND METHODS

### 2.1 Materials and chemicals

WPI (>90%) was purchased from Xi'an Zhuorui Biotechnology Co., Ltd and C3G (mulberry) (95% purity) from Yuanye Bio‐Technology Co., Ltd. (Shanghai, China). Formic acid, methanol, hydrochloric acid (HCl), and sodium hydroxide (NaOH) were purchased from Chemical Reagent Co., Ltd. (Shanghai, China). All solutions were freshly prepared before use.

### 2.2 Preparation of the stock solution

Dissolve WPI powder in a phosphate buffer solution at pH 7.0 (10 mmol/L) to create a WPI stock solution (1 mg/mL). To ensure full hydration, use a magnetic stirrer to gently agitate the solution at 25°C for 3 h. Then, refrigerate the solution overnight at 4°C. The pH 7.0 phosphate buffer solution was employed to dissolve the C3G powder, to prepare C3G stock solutions of various concentrations (0, 10, 20, 30, 40, and 50 μg/mL), to mix a certain volume of WPI with C3G solution to form a C3G–WPI mixture and to then gently stir in the dark at 25°C for 2 h. Thereafter, the mixtures were kept at 4°C until analyzed. The C3G and WPI solution of each experiment was freshly prepared.

### 2.3 Ultraviolet–visible absorption spectroscopy measurement

Blending certain protein solutions and C3G concentrations resulted in a final WPI solution concentration of 0.1 mg/mL and final C3G concentrations of 0, 10, 20, 30, 40, and 50 μg/mL at pH 7.0. The ultraviolet (UV)–visible spectrum of absorption was scanned within 200 and 500 nm employing an ultraviolet (UV) spectrometer (Beijing Shimadzu Medical Instrument Co., Ltd., Beijing, China). All experiments were repeated three times at room temperature.

### 2.4 Fluorescence spectroscopy measurement

By employing a fluorescence spectrophotometer (F‐2700, Hitachi, Japan) set at 298, 308, and 318 K, measure the fluorescence intensity of C3G on WPI. The protein solution and C3G solution were mixed at pH 7.0, resulting in a final concentration of 0.1 mg/mL for the WPI solution and 10, 20, 30, 40, and 50 μg/mL for the C3G solution. The excitation wavelength was 280 nm, emission wavelength 300–450 nm, and the slit width was 5 nm, and the voltage 700 volts. In the emission wavelength range of 220–600 nm, the fluorescence spectra of each mixed system were collected using a four‐way micro cuvette (1 mm slit), the path length of the cuvette 1 cm. During the calculation of fluorescence parameters, the background fluorescence values of the corresponding compounds (fluorescence spectra of C3G with phosphate buffer solution) were recorded and deducted. The quenching mechanisms were analyzed using the Stern–Volmer (Ma et al., 2022) and double logarithmic regression equations (Cheng et al., 2017).

(1) $$\frac{F_{0}}{F}=1+K_{\mathrm{SV}}\left[Q\right]=1+K_{q}\tau_{0}\left[Q\right]$$

(2) $$\mathrm{lg}\frac{F_{0}-F}{F}=\mathrm{lg}K_{a}+n\mathrm{lg}\left[Q\right]$$

where the C3G‐containing and C3G‐free proteins’ fluorescence intensities are denoted by *F* and *F*_0_, respectively; *K*_SV_ is a dynamic Stern–Volmer quenching constant; and *K*_q_ is a bimolecular quenching rate constant controlled by diffusion. [*Q*] represents the concentration of C3G; *τ*_0_ represents the average lifespan of fluorescent molecules in the absence of C3G, and the fluorescence lifetime of biological macromolecules is around (10^−8^ s). The binding constants and the number of binding sites between C3G and WPI are indicated by *K*_a_ and *n*, respectively. The thermodynamic parameters were computed using the Van't Hoff equation.

(3) $$\ln K_{a}=-\frac{∆H}{R}\times \frac{1}{T}+\frac{∆S}{R}$$

(4) $$∆G=∆H-T∆S=-\mathrm{RT}\ln K_{a}$$

where ∆*H*, ∆*S*, and ∆*G* are defined as changes in the enthalpy, entropy, and free energy, respectively. *T* represents the absolute temperature; and *R* is the gas constant (8.314 J/mol/K).

In addition, at 298 K, the concentration of the solution in synchronous fluorescence spectrum is consistent with the concentration of the solution in the fluorescence spectrum, and the interval between the excitation and emission wavelengths is fixed at 15 and 60 nm (∆).

### 2.5 Secondary structure measurement

The secondary structures of the WPI and C3G–WPI mixtures were determined by Raman spectroscopy (HORIBA XploRA PLUS). The final concentrations of 1 mg/mL for WPI and 0 and 0.5 mg/mL for C3G at pH 7.0 were obtained by blending specific protein solutions and C3G concentrations. The sample solution was drawn into a glass capillary. The laser wavelength was set at 785 nm, with a laser power of 100 mW. The scanning range spanned 400–3600 cm^−1^, with a spectral resolution of 2.0 cm^−1^. The procedure involved scanning the sample three times, with 60 exposures, acquiring data at 1 cm^−1^, and a scanning speed of 120 cm^−1^/min. PeakFit 4.12 was utilized to analyze the protein amide I band (1600–1700 cm^−1^), involving baseline correction, deconvolution, and second derivative fitting. The relative contents of WPI's secondary structure were calculated based on the area of each peak.

### 2.6 Thermal stability test

Refer to the method provided by Attaribo et al. (2021) for thermal stability analysis. At pH 7.0, appropriate solutions of WPI and C3G were combined to yield concentrations of 0.1 mg/mL WPI and 0.05 mg/mL C3G. The sample solutions were heated in a water bath in the dark at 80 and 95°C for durations of 0, 20, 40, 60, and 80 min before being rapidly cooled. After heat processing, 5 mL of pH buffer was used to dilute the solutions for determining the concentration of C3G. An ultraviolet (UV) spectrophotometer was used to measure the absorbance at 520 nm. Heat degradation kinetics were investigated using first‐order kinetic equations. At the treatment temperature, the half‐life (*T*_1/2_) was calculated as the time when the C3G concentration decreased to 50% of its original concentration.

(5) $$C_{t}=e^{-\mathrm{kt}}\cdot C_{0}$$

(6) $$T_{1/2}=-\frac{\ln\left(0.5\right)}{K}$$

where *K* is the degradation rate constant, *C*_0_ and *C*_*t*_ are the initial C3G concentrations, and heating for *t* min at 85 and 95°C.

### 2.7 Oxidation stability test

Conduct oxidation tests on C3G–WPI mixtures and C3G only samples. Freshly prepare 0.15 mg/mL C3G solution and 0.15 mg/mL WPI solution using pH 7.0 phosphate buffer, and prepare C3G–WPI mixtures in a 1:1 volume ratio for oxidative stability testing. In order to perform the oxidation stability test, the sample is mixed with a final concentration of 0.05 mg/mL of hydrogen peroxide (H_2_O_2_) and allowed to oxidize for 1 h at room temperature in the dark. Afterwards, the oxidized samples are subjected to further analysis.

### 2.8 Measurement of color

To compare the colors of oxidized and untreated samples, use a 3NH colorimeter (Shenzhen Sanenshi Technology Co., Ltd., China) and a quartz colorimetric cup (2 mL sample) with a path length of 0.5 cm. The results designated by L* (brightness), a* (red green), and b* (yellow blue) are used to assess the sample's color change. The color change (∆*E*) is obtained using Equation (7). To assess the protective impact of WPI on C3G, compare the oxidized C3G sample to the oxidized sample with WPI, using untreated C3G as a control.

(7) $$∆E=\sqrt{{\left(L^{*}-L_{0}^{*}\right)}^{2}+{\left(a^{*}-a_{0}^{*}\right)}^{2}+{\left(b^{*}-b_{0}^{*}\right)}^{2}\;}$$

where *L*_0_*, *a*_0_*, and *b*_0_* are the color values of the untreated C3G solution (0.15 mg/mL).

### 2.9 Data analysis

All tests were conducted in triplicate and analyzed using SPSS 25.0 statistical software to determine the difference between the mean values, with a significance level of *p* < .05. Origin was used to create the graphs (2023).

## 3 RESULTS AND DISCUSSION

### 3.1 Ultraviolet–visible spectroscopy analysis

**FIGURE 1:**
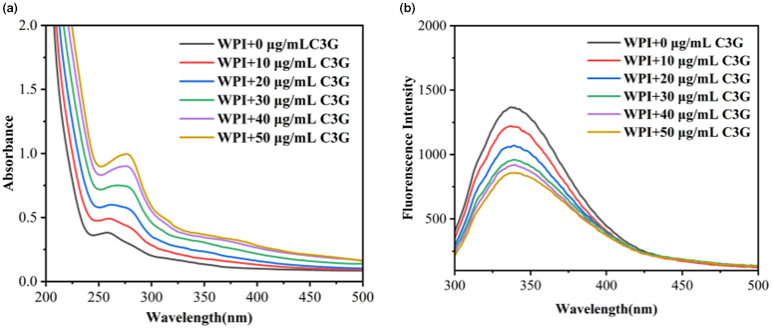
Ultraviolet (UV)–visible absorption spectra of WPI with or without C3G (a). Fluorescence spectra of WPI with or without C3G (b).

Ultraviolet (UV)–visible absorption spectroscopy is frequently used to analyze the interaction between tinycompounds and biological macromolecules (Huo et al., 2019; Li et al., 2023). The interaction between C3G and WPI was preliminarily judged by comparing the similarities and differences in the ultraviolet (UV)–visible absorption spectra of WPI upon adding different concentrations of C3G (0, 10, 20, 30, 40, and 50 μg/mL). As shown in Figure 1a, the (UV)–visible spectrum of WPI exhibited a smooth curve, and its absorption value increased as the concentration of C3G increased. Compared with the WPI without C3G, the maximum absorption peak of the WPI with C3G was significantly redshifted (from 258 to 277 nm), demonstrating that the interaction with C3G altered the walnut protein's spatial structure. This change was similar to the structural changes observed in the silkworm pupa protein–glucose conjugate when it interacted with C3G (Attaribo et al., 2021), the peak uptake of the silkworm pupa protein–glucose copolymer increased gradually with increasing C3G concentration, suggesting that anthocyanins interacted with the protein. The above results indicated that the C3G interacted with WPI, which might cause the extension of the peptide chain and produce a new conformation.

### 3.2 Fluorescence spectroscopy analysis

Fluorescence spectroscopy was often employed to analyze the interaction mechanism between proteins and polyphenols (Li et al., 2024). Figure 1b demonstrates the effects of various C3G concentrations (0, 10, 20, 30, 40, and 50 μg/mL) on the WPI fluorescence spectra. The fluorescence intensity of WPI interacting with C3G was lower than that of WPI alone at the fluorescence spectra between 340 and 350 nm, showing that the interaction of C3G and WPI reduced WPI's intrinsic fluorescence. However, the fluorescence signal of C3G itself is very weak, and the interference in the fluorescence signal of WPI can be ignored. Therefore, this study does not need to consider the interference problem of “internal filtering effect.” The maximum fluorescence emission peak was slightly redshifted (from 337 to 339 nm) after the addition of C3G, indicating that the C3G binding altered the chromophore's amino acid microenvironment. Tyrosine (Tyr) and tryptophan (Trp) residues may have directly interacted with C3G, causing a quenching of WPI's fluorescence. Ren et al. (2019) also found a similar phenomenon in the interaction between resveratrol and trypsin, the interaction between resveratrol and trypsin caused a redshift in the absorption peak of the trypsin fluorescence spectrum, resulting in a change in the microenvironment around the tyrosine and tryptophan residues of trypsin.

### 3.3 Fluorescence quenching type analysis

**FIGURE 2:**
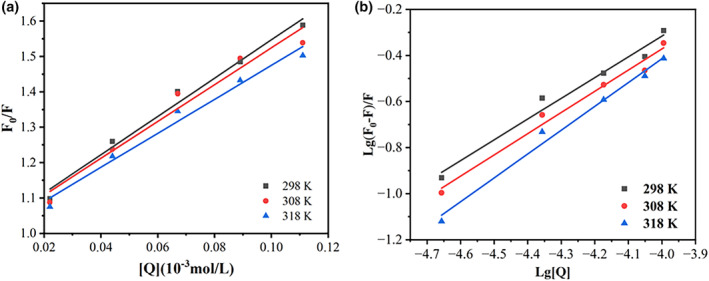
Stern–Volmer plots of *F*_0_/*F* with different C3G concentrations at different temperatures of 298, 308, and 318 K (a). Lg [(*F*_0_−*F*)/*F*] plots of different C3G concentrations at different temperatures of 298, 308, and 318 K (b). Free amino acid content of different fermented Tongling white ginger.

Fluorescence quenching was commonly classified into two types: dynamic and static (Chen et al., 2021). The fluorescence quenching behavior of C3G on WPI at different temperatures was studied using the Stern–Volmer equation. An excellent linear relationship was observed at different concentrations of C3G (0, 10, 20, 30, 40, and 50 μg/mL) (Figure 2a), which indicated the existence of a single quenching mechanism. The value of *K*_sv_ steadily decreased as the temperature increased (Table 1). At the same time, the *K*_q_ values were higher than the maximal diffusion constant of collision quenching (2 × 10^10^ L/mol/s), suggesting that static quenching was the primary mechanism of the interaction between C3G and WPI.

**TABLE 1:** The thermodynamic parameters, binding constants, binding sites, molecular quenching constants (*K*_q_), and Stern–Volmer quenching constants (*K*_SV_) for the interactions of C3G with the WPI system at 298, 308, and 318 K.

System	*T* (K)	*K*_SV_ (10^3^ L/mol)	*K*_q_ (10^11^ L/mol/s)	*n*	*K*_a_ (10^3^ L/mol)	∆*H* (KJ/mol)	∆*S* (J/mol/K)	∆*G* (kJ/mol)
C3G–WPI	298	5.42 ± 0.062^a^	5.42 ± 0.062^a^	0.9 ± 0.08^a^	1.46 ± 0.078^c^	18.62	89.5	−8.05 ± 0.08^c^
	308	5.14 ± 0.037^b^	5.14 ± 0.037^b^	0.92 ± 0.07^a^	1.69 ± 0.019^b^			−8.47 ± 0.05^b^
	318	4.58 ± 0.085^c^	4.58 ± 0.085^c^	1.03 ± 0.06^a^	1.91 ± 0.038^a^			−9.84 ± 0.07^a^

*Note*: The superscripts (a–c) represents significant differences (*p* < 0.05).

The double logarithmic regression curve of the interaction between GSP and WPI is shown in Figure 2b. Calculating the slope of the curve revealed that the binding constant *K*_a_ values were all more than 103 at 298, 308, and 318 K, showing a strong affinity between C3G and WPI. At the above temperature, the *n* value is approximately 1, indicating that they only have one binding site and form a complex with an approximate ratio of 1:1. These results indicated that C3G could bind to the vicinity of Trp and/or Tyr residues (the intrinsic fluorophores of WPI), which induces the fluorescence quenching of WPI.

The interaction between ligands and biomacromolecules can be evaluated using the enthalpy change value (Δ*H*) and entropy change value (Δ*S*). Noncovalent interactions between ligands and macromolecules include hydrophobic, hydrogen bonding, electrostatic, and van der Waals forces (Roufegarinejad et al., 2018). The resulting thermodynamic parameters are shown in Table 1. The spontaneous nature of the binding process between WPI and C3G is demonstrated by Δ*G* < 0. The positive ΔH indicates that the interaction between C3G and WPI is an endothermic process. During the combination of C3G and WPI, the computed values for Δ*H* and Δ*S* were 18.62 KJ/mol and 89.5 J/mol/K, respectively. The presence of hydrophobic interactions as the primary driving force in the binding reaction is suggested when Δ*H* > 0 and Δ*S* > 0 (He et al., 2016b), indicating that hydrophobic forces play an essential part in the interaction between C3G and WPI. The results of the interaction between thymol and bovine serum albumin were comparable to the observation results shown in Table 1.

### 3.4 Synchronous fluorescence spectrum analysis

**FIGURE 3:**
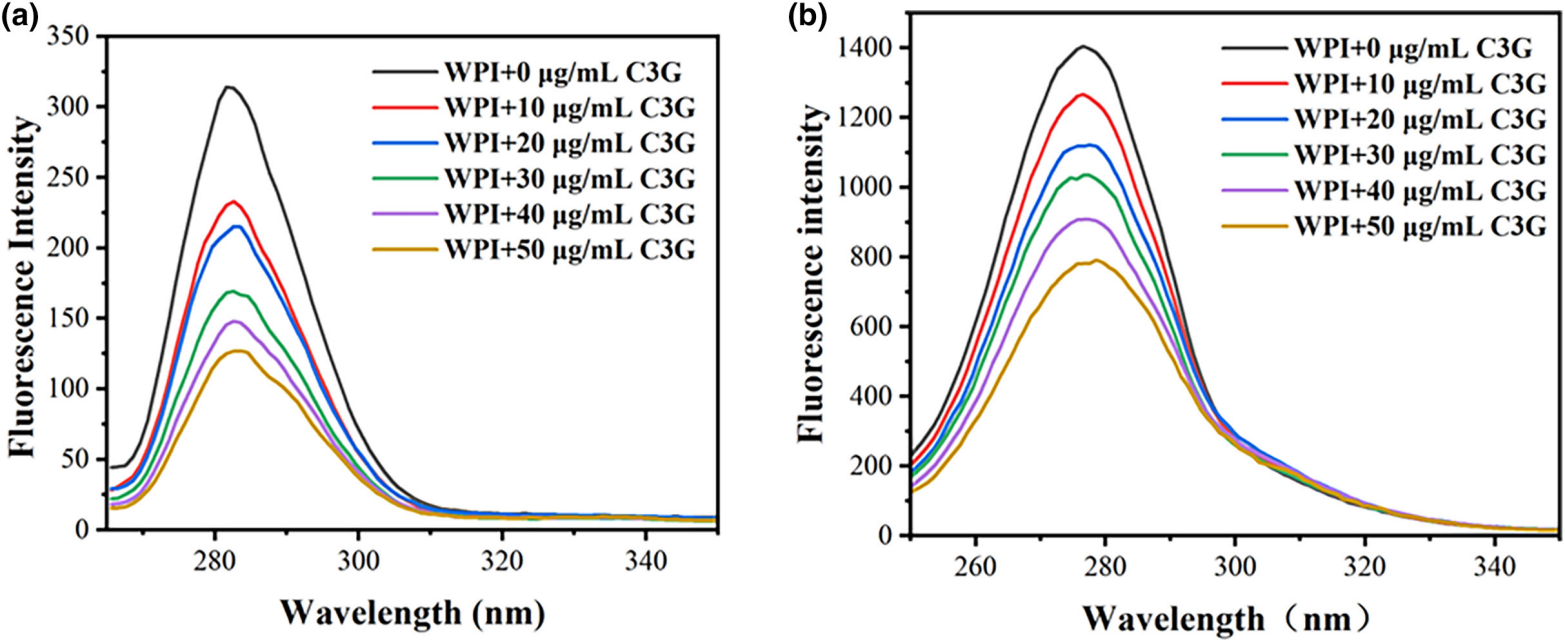
Synchronous fluorescence spectra of WPI with and without C3G at 298 K. ∆*λ* = 15 nm (a). ∆*λ* = 60 nm (b) and the concentrations of C3G and WPI are consistent with the fluorescence.

Synchronous fluorescence spectrometry was employed to investigate the impact of C3G on the amino acid residues' microenvironment in WPI (Tao et al., 2022). When ∆*λ* was 15 nm, only the fluorescence characteristic spectrum of Tyr residues is displayed. When ∆*λ* was 60 nm, only the distinctive fluorescence spectrum of Trp residues can be observed (Jing et al., 2014). As a result, changes in the maximum emission wavelength can be utilized to evaluate the polarity shift of the microenvironment surrounding the protein's Tyr and Trp residues. As shown in Figure 3, the increasing concentration of C3G gradually reduced the synchronized fluorescence intensities of both Tyr and Trp, indicating that both residues are involved in the interaction. The maximum emission wavelength of Tyr residue shows a slight redshift (281–283 nm), indicating a decrease in hydrophobicity and an increase in polarity around the Tyr residue of WPI (Figure 3a). The tyrosine residue gradually becomes exposed with increasing C3G concentration (Yu et al., 2020). However, no discernible redshift was observed for the Trp residue (Figure 3b), indicating that there were no significant changes to the polarity microenvironment of tryptophan. Additionally, the binding site and the Tyr residue are closer. Similar results were observed from the interactions of C3G with whey protein and black bean isolate protein (Gong et al., 2021; Wang & Xie, 2019).

### 3.5 Secondary structure analysis

**FIGURE 4:**
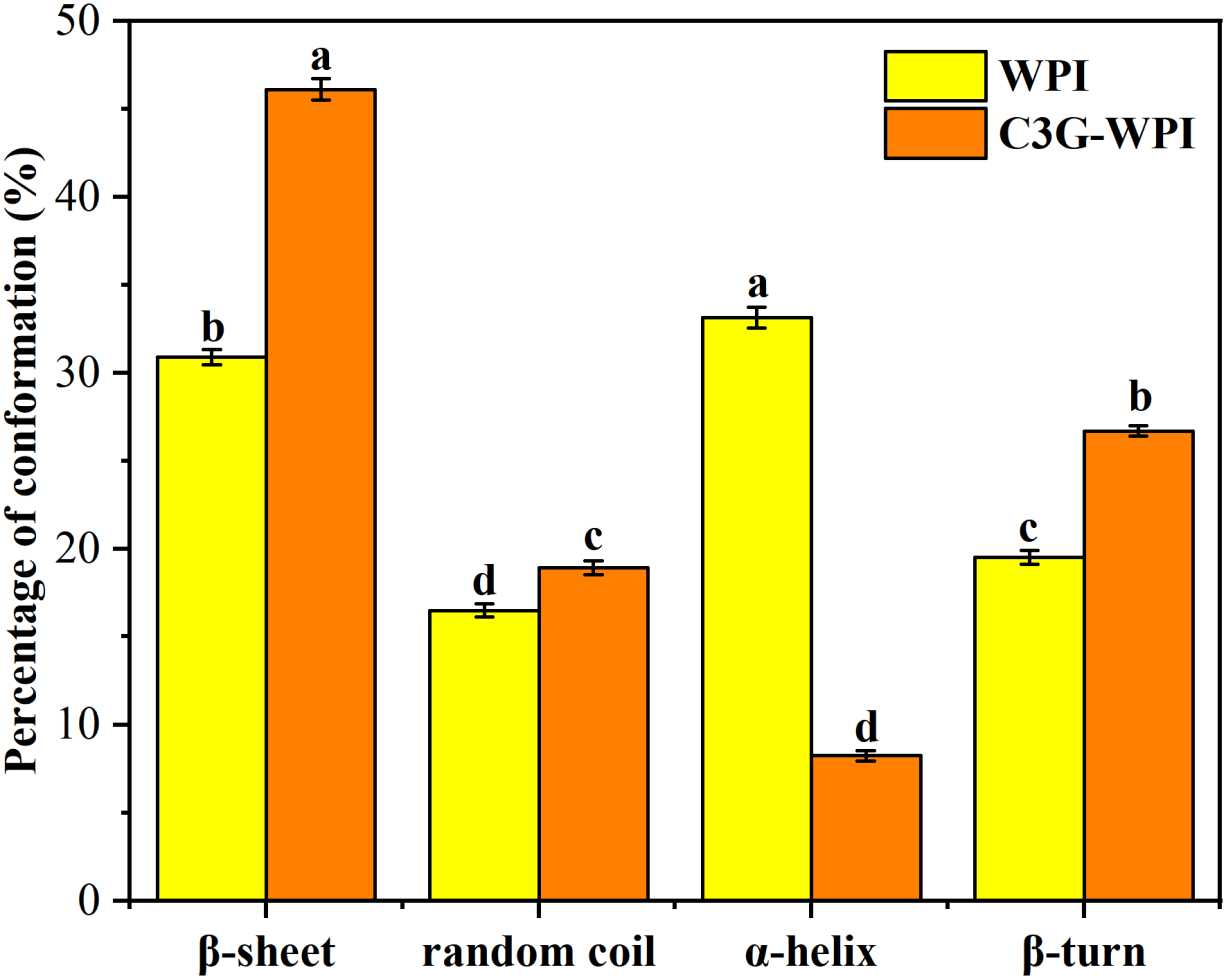
Percentage of conformation of WPI with or without C3G at 298 K, a–d represents significant differences (*p* < .05).

Raman spectroscopy was an effective method for identifying protein conformational changes following ligand binding (Huang et al., 2006). PeakFit 4.1.2 was used to estimate the secondary structure content of the WPI and C3G–WPI mixtures. Generally, the peak positions of the amide I band in the 1600–1700 cm^−1^ spectrum area of proteins are primarily composed of antiparallel β‐sheets (1680–1690 cm^−1^), α‐helix (1650–1660 cm^−1^), random coils (1640–1650 cm^−1^), and secondary structural β‐sheets (1610–1640 cm^−1^) (Gui et al., 2023). As shown in Figure 4, the α‐helix content significantly decreased from 33.13% to 8.27% when WPI bound to C3G (*p* < .05). Meanwhile, there was an increase in β‐sheet content (from 30.89% to 46.12%) (*p* < .05), β‐turn content (from 19.5% to 26.69%) (*p* < .05), and random coil content (from 16.48% to 18.92%) (*p* < .05). A similar phenomenon was also observed in the structural alteration of trypsin induced by resveratrol binding (Ren et al., 2019). The secondary structure of WPI becomes looser and more extended when the α‐helix content decreases and β‐sheet content increases. Protein polarity increased and hydrophobicity decreased as polar groups became exposed. These findings corroborate the results of the fluorescence tests.

### 3.6 Thermal stability analysis

**FIGURE 5:**
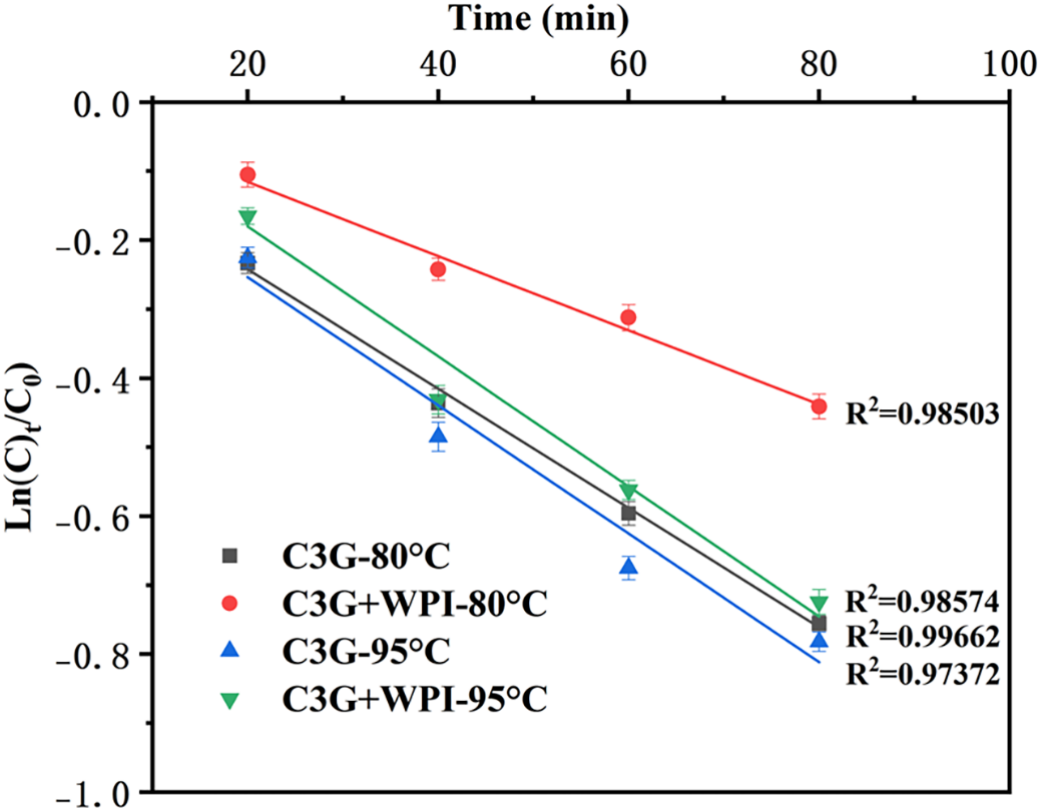
The degradation kinetics of C3G (0.05 mg/mL) with and without WPI (0.1 mg/mL) at 80 and 95°C for 20, 40, 60, and 80 min.

The degradation kinetics of C3G and C3G–WPI mixtures at 80 and 95°C are shown in Figure 5. The correlation coefficients (*R* ^2^) were all above 0.97, demonstrating that the degradation kinetics of C3G, whether or not it included WPI, corresponded to first‐order kinetics (Attaribo et al., 2021; Ma & Jing, 2020). The results for the degradation rate constant *K* and the half‐life *t*_1/2_ are shown in Table 2. The C3G degradation rate constants *K* were 0.00865 and 0.0093, and the half‐lives were 80.18 and 73.76 min, respectively. After interacting with WPI, the half‐life of C3G significantly increased from (80.18 ± 1.86) min to (129.8 ± 9.67) min at 80°C (*p* < .05), at 95°C, it was prolonged from (73.76 ± 9.75) min to (74.12 ± 6.3) min (*p* > .05). Additionally, there was a decrease in *K*'s degradation rate constant. The outcomes showed that adding WPI increased the thermal stability of anthocyanin in a heating environment. Whey protein isolate and black soybean protein isolate have also been reported to improve anthocyanin thermal stability (Chen et al., 2022; Wang & Xie, 2019), whey protein isolate could reduce the degradation rate of anthocyanins by 18.1% at 80°C for 120 min, and black soybean protein isolate could increase the half‐life of anthocyanins by 2.5 and 1.16 times at 85 and 100°C for 80 min. In this study, WPI improved the half‐life of anthocyanin by 2.2 and 1.3 times at 80 and 95°C, respectively. As a result, WPI was found to be an excellent plant protein source that may protect C3G against degradation.

**TABLE 2:** Degradation reaction rate constants (*K*) and half‐life (*T*_1/2_) of C3G with and without WPI.

Samples	*K* (min^−1^)	*T*_1/2_ (min)
C3G‐80°C	0.00865 ± 0.0002^a^	80.18 ± 1.86^b^
C3G–WPI‐80°C	0.00537 ± 0.0004^b^	129.8 ± 9.67^a^
C3G‐95°C	0.00930 ± 0.00108^a^	73.76 ± 9.75^b^
C3G–WPI‐95°C	0.00942 ± 0.0008^a^	74.12 ± 6.3^b^

*Note*: The superscripts (a,b) represents significant differences (*p* < 0.05).

### 3.7 The effects of WPI on the oxidation stability of C3G

The effects of WPI on the oxidation stability of the C3G at pH 7.0 are shown in Table 3. The *L*_0_*, *a*_0_*, and *b*_0_* values of C3G were 17.18, 18.01, and 12.88, respectively. Compared with the *L*_0_*, *a*_0_*, and *b*_0_* values, the a* and b* values of the oxidized C3G samples (0.05% H_2_O_2_ for 1 h) decreased (*p* < .05), while their L* values increased (*p* < .05), suggesting that H_2_O_2_ oxidation caused color loss in the C3G samples. After H_2_O_2_ oxidation, C3G mixed with WPI has a lower ∆E value (*p* < .05), but higher a* value and overall C3G content than C3G without WPI. At pH 7.0, C3G breakdown rates were 57.2% in a mixture of C3G and WPI treated with oxidation. The degradation rate is significantly lower than that of samples containing only C3G (70.3%) (*p* < .05). In summary, the addition of WPI effectively prevented color loss and degradation of C3G samples during the oxidation process at pH 7.0. Casein and whey proteins also have similar protective effects on grape skin anthocyanin extract solutions (He et al., 2016a).

**TABLE 3:** Effect of WPI on colorimetric parameters, total C3G contents, and degradation rate of C3G solutions after oxidation test (0.05% H_2_O_2_ for 1 h) at pH 7.0.

Samples	L*	a*	b*	∆*E*	Total C3G contents (mg/L)	Degradation rate of C3G (%)
C3G	17.18 ± 0.06^c^	18.01 ± 0.07^a^	12.88 ± 0.12^a^	0.00 ± 0.00^b^	138.00 ± 0.13^a^	0.00 ± 0.00^c^
C3G (H_2_O_2_)	28.91 ± 0.09^b^	7.29 ± 0.04^c^	3.56 ± 0.13^b^	18.42 ± 0.03^a^	43.00 ± 0.35^c^	70.30 ± 0.30^a^
C3G + WPI (H_2_O_2_)	29.60 ± 0.14^a^	10.85 ± 0.38^b^	1.68 ± 0.12^c^	18.26 ± 0.15^a^	62.00 ± 0.78^b^	57.20 ± 0.50^b^

*Note*: The superscripts (a–c) represents significant differences (*p* < 0.05).

## 4 CONCLUSION

In this study, multiple spectroscopic techniques were utilized to analyze the interaction between C3G and WPI, and found that hydrophobic interactions were the primary driving factor promoting spontaneous reaction. The quenching mechanism is a static quenching caused by the formation of a nonluminescent ground state complex between C3G and WPI. Synchronous fluorescence revealed that the C3G and WPI binding sites were close to the Tyr residue. The secondary structure of WPI changes after interacting with C3G, α‐helix content decreased from 33.13% to 8.27%, and β‐sheet content increased from 30.89% to 46.12%. Degradation kinetics study results showed that the degradation of C3G at 80 and 95°C followed first‐order kinetics. The half‐life of C3G was extended from 80.18 to 129.8 min at 80°C and from 73.76 to 74.12 min at 95°C after interacting with WPI. Furthermore, at pH 7.0, the addition of WPI effectively increased the oxidation stability of C3G. These findings elucidate the modes of interaction between C3G and WPI and demonstrate the enhanced thermal and oxidation stability of C3G after interaction with WPI, indicating that WPI is a potential stabilizer, and it expands the application range of C3G in the field of food processing.

Due to the absence of the molecular structure of WPI in Protein Data Bank (PDB), this study was unable to provide a clear molecular‐level explanation for the mechanism by which the interaction between WPI and C3G enhances the stability of C3G. This leads to a lack of in‐depth understanding of the mechanism by which WPI enhances C3G stability. To make up for this deficiency, we plan to conduct detailed characterization of the structure of WPI in subsequent research and use molecular docking technology to clarify the protective mechanism of WPI on C3G stability. This molecular‐level analysis will help reveal the interaction mechanism between WPI and C3G, providing important theoretical support for further optimizing the stability of C3G in food.

## AUTHOR CONTRIBUTIONS

**Daquan Wang:** Conceptualization (equal); data curation (equal); formal analysis (equal); investigation (equal); methodology (equal); software (equal); validation (equal); writing – review and editing (equal). **Haipeng Cui:** Data curation (equal); formal analysis (equal); investigation (equal); validation (equal). **Kaili Zong:** Formal analysis (equal); investigation (equal); methodology (equal); validation (equal). **Hongchao Hu:** Data curation (equal); investigation (equal); supervision (equal). **Yali Li:** Formal analysis (equal); investigation (equal); supervision (equal). **Jianting Yang:** Funding acquisition (equal); resources (equal); supervision (equal); writing – review and editing (equal).

## FUNDING INFORMATION

This study was funded by the Key Scientific Research Project of Anhui Provincial Department of Education (Project No. 2023AH051868).

## CONFLICT OF INTEREST STATEMENT

The authors have no conflict of interest to declare.

## Data Availability

The data that support the findings of this study are available from the corresponding author upon reasonable request.
